# CRISPR-Cas9 knockout of membrane-bound alkaline phosphatase or cadherin does not confer resistance to Cry toxins in *Aedes aegypti*

**DOI:** 10.1371/journal.pntd.0012256

**Published:** 2024-06-13

**Authors:** Sabino Pacheco, Adrián S. Gallegos, Ángel E. Peláez-Aguilar, Jorge Sánchez, Isabel Gómez, Mario Soberón, Alejandra Bravo

**Affiliations:** Departamento de Microbiología Molecular, Instituto de Biotecnología, Universidad Nacional Autónoma de México (UNAM), Cuernavaca, Morelos, Mexico; Kenya Agricultural and Livestock Research Organization, KENYA

## Abstract

The *Aedes aegypti* cadherin-like protein (Aae-Cad) and the membrane-bound alkaline phosphatase (Aae-mALP) are membrane proteins identified as putative receptors for the larvicidal Cry toxins produced by *Bacillus thuringiensis* subsp. *israelensis* bacteria. Cry toxins are the most used toxins in the control of different agricultural pest and mosquitos. Despite the relevance of Aae-Cad and Aae-mALP as possible toxin-receptors in mosquitoes, previous efforts to establish a clear functional connection among them and Cry toxins activity have been relatively limited. In this study, we used CRISPR-Cas9 to generate knockout (KO) mutations of Aae-Cad and Aae-mALP. The Aae-mALP KO was successfully generated, in contrast to the Aae-*Cad* KO which was obtained only in females. The female-linked genotype was due to the proximity of *aae-cad* gene to the sex-determining *loci* (*M*:*m*). Both *A*. *aegypti* KO mutant populations were viable and their insect-development was not affected, although a tendency on lower egg hatching rate was observed. Bioassays were performed to assess the effects of these KO mutations on the susceptibility of *A*. *aegypti* to Cry toxins, showing that the Aae-Cad female KO or Aae-mALP KO mutations did not significantly alter the susceptibility of *A*. *aegypti* larvae to the mosquitocidal Cry toxins, including Cry11Aa, Cry11Ba, Cry4Ba, and Cry4Aa. These findings suggest that besides the potential participation of Aae-Cad and Aae-mALP as Cry toxin receptors in *A*. *aegypti*, additional midgut membrane proteins are involved in the mode of action of these insecticidal toxins.

## Author summary

The Cry toxins produced by the bacterium *Bacillus thuringiensis* subsp. *israelensis* have been used for decades to control populations of mosquito responsible for spreading febrile diseases in tropical regions. These insecticidal toxins specifically target the midgut cells of larvae. It is widely accepted that two membrane proteins, cadherin (Aae-Cad) and membrane-bound alkaline phosphatase (Aae-mALP), are receptors for Cry toxins in *A*. *aegypti*. With the aim to study the functional role of Aae-Cad and Aae-mALP in the mode of action of mosquitocidal Cry toxins, in this study we employed the CRISPR-Cas9 system to generate knockout *A*. *aegypti* strains deficient in either Aae-Cad or Aae-mALP. Interestingly, we found a sex-linked association for mutations in Aae-Cad generated by CRISPR-Cas9, attributable to its proximity to the sex-determining M-factor on chromosome 1. Furthermore, the CRISPR-Cas9-mediated knockout of Aae-Cad or Aae-mALP proteins did not affect the normal development of the insects. Surprisingly, we found that the absence of Aae-Cad in the KO-females or Aae-mALP KO *A*. *aegypti* did not generate a resistance phenotype to four different Cry toxins, Cry11Aa, Cry11Ba, Cry4Ba and Cry4Aa, indicating that additional molecules are required in the mode of action of Cry toxins.

## Introduction

*Bacillus thuringiensis* subsp. *israelensis* (Bti) is globally recognized as a specific agent for the biological management of dipteran insects. Notably, Bti is highly effective against the mosquito *Aedes aegypti*, considered the primary vector of viruses responsible for fever diseases in humans, such as dengue, Zika, and chikungunya, which are prevalent in tropical and subtropical regions. The insecticidal activity of Bti relies on the parasporal crystalline inclusions that are produced during the sporulation phase of growth, which are composed of insecticidal proteins, including three Cry toxins, Cry4Aa, Cry4Ba and Cry11Aa and one Cyt toxin, Cyt1Aa [1]. These toxins are pore-forming proteins that target the larval midgut cells. The high specificity of Cry toxins is mostly given by specific interactions with insect-proteins that are localized on the apical microvilli of the midgut cells that act as toxin-receptors. Upon binding, Cry toxins form oligomers that insert into the membrane forming lytic pores that breakdown the integrity of gut epithelium, finally leading to the insect death [2]. Multiple membrane proteins have been identified in mosquitoes as putative receptors for Cry toxins, including cadherin-like proteins (Cad) and glycosylphosphatidylinositol (GPI)-anchored proteins, such as alkaline phosphatase (ALP) and aminopeptidase-N (APN) [3].

The Cad protein was first identified in the lepidopteran *Manduca sexta* as putative receptor for Cry1Ab toxin [4]. Disruptive mutations of the *cad* gene frequently confer resistance to Cry1 toxins in different Lepidopteran larvae such as *Heliothis virescens*, *Pectinophora gossypiella* and *Helicoverpa armigera* [5–7]. Further characterization showed that Cad plays an important role in the mode of action, facilitating the oligomerization of Cry1Ab toxin [8]. In *A*. *aegypti*, the Aae-Cad was also identified as a putative receptor of Cry11Aa [9], while the *Anopheles gambiae* Ag-Cad1 was proposed as a receptor for Cry4Ba, and Ag-Cad2 was identified as receptor for Cry11Ba toxin, which is produced by *B*. *thuringiensis* subsp. *jegathesan* (Btj) [10,11]. *In vitro* binding assays have demonstrated that recombinant fragments of Aae-Cad exhibit high-affinity binding to Cry11Aa. The Cry11Ba and Cry4Aa toxins, in contrast to Cry4Ba, are also able to bind with Aae-Cad, suggesting that Aae-Cad may also serve as a receptor for Cry11Ba and Cry4Aa toxins in *A*. *aegypti* [9]. Silencing Aae*-*Cad gene by double stranded RNA interference (dsRNAi) showed that the silenced larvae become tolerant to Cry11Aa but not to Cry4Ba, supporting that Aae-Cad plays a role as a receptor for Cry11Aa toxin but not for Cry4Ba. Furthermore, it was previously demonstrated that the oligomerization of Cry11Aa toxin relies on binding with Aae-Cad, whereas Cry4Ba could form oligomers even in the absence of Aae-Cad [12].

In the case of ALP, two Cry-binding ALPs have been characterized as putative receptors in the mosquito *A*. *aegypti*. The GPI-anchored ALP1 isolated from the midgut tissue brush border membrane vesicles (BBMV) was firstly identified as a Cry11Aa-binding protein [13]. Further studies showed that dsRNAi silencing of ALP1 in *A*. *aegypti* larvae confers tolerance to Cry11Aa and Cry11Ba [14]. Similar results were observed when Cry4Ba was tested with dsRNAi ALP1-silenced *A*. *aegypti* larvae, although the tolerance was lower in comparison to Cry11Aa [15]. Moreover, another membrane-bound ALP (mALP) was identified from *A*. *aegypti* BBMV as Cry4Ba-binding protein [16]. Heterologous expression of Aae-mALP in the *Spodoptera frugiperda* Sf9 cell line confers susceptibility to Cry4Ba toxin, suggesting that Aae-mALP is a functional receptor for Cry4Ba toxin [17]. However, *A*. *aegypti* contains several ALPs and their role as functional receptors is complex. At least six ALPs were identified by a proteomic approach showing that they were associated to lipid rafts and were proposed as potential Cry4Ba-binding receptors, among these ALP’s the Aae-mALP was identified but not the ALP1 [18]. On the other hand, a transcriptomic study performed in *A*. *aegypti* populations that developed resistance to Bti toxins showed no significant up/down-regulation differences in the expression of 11 ALPs proteins in these resistant strains [19]. The analysis comparing expression of ALP proteins in different *A*. *aegypti* strains selected for their resistance to different Cry toxins (resistant to Cry4Aa, Cry4Ba, Cry11Aa, and Bti) showed differential expression patterns of ALP clusters, depending on the resistance to the individual toxins or Bti crystal, suggesting that the resistance might be a multigenic phenomenon.

This work is focused on studying the functional role of Aae-Cad and Aae-mALP on Cry toxin activity by using the CRISPR-Cas9 system to generate knockout mutations of these genes in *A*. *aegypti*. The study delves into the repercussions of these KO mutations on the development of *A*. *aegypti* and their susceptibility to different mosquitocidal Cry toxins.

## Materials and methods

### Rearing of *Aedes aegypti*

The *A*. *aegypti* laboratory population has been stablished for over 15 years at Instituto de Biotecnología-UNAM facilities. *A*. *aegypti* wild-type and CRISPR-Cas9 edited populations were maintained at 28°C, 75–80% humidity with a photoperiod of 12h:12h (dark: light). Larvae were fed with commercial dog food and developed in tap water. Adults were fed with a solution of 10% honey bee. Adult females were fed with cattle blood supplemented with 1.5 mg/ml of EDTA, contained in a petri dish sealed with a Parafilm^M^ membrane (Sigma-Aldrich).

### Design and synthesis of sgRNA

Exon and intron regions of *aae*-*malp* (AAEL015070) and *aae-cad* (AAEL024535) genes were identified from the assembled genome AaegL5.3 of *A*. *aegypti* (www.vectorbase.org) and the 5’ exons were considered for prediction of single-guide RNA (sgRNA) using the web tool CRISPOR [20]. Two sgRNAs were designed per gene, sgRNA-55rv and sgRNA-114fw targeted the third exon of *aae-cad*, while sgRNA-23fw and sgRNA-124rv targeted the first exon of aae-*malp* (Table 1). *In vitro* synthesis of these sgRNAs was performed by assembly of two overlapping primers by PCR with Phusion Hight-Fidelity DNA polymerase (Thermo Scientific). The two primers were: a specific forward primer for each sgRNA and the common reverse primer T7common (Table 1). Purified PCR product was used as template for *in vitro* transcription using the TranscriptAid T7 High Yield Transcription kit (Thermo Scientific). Finally, the sgRNA were purified with the RNA Clean and Concentrator kit (Zymo Research).

**Table 1:** Primers for synthesis of sgRNA, PCR and RT-PCR.

Primer	Sequence 5’➔3’
sgRNA-55rv	GAAATTAATACGACTCACTATAGGCTGCTCCCATTCCCCTGGGTTTTAGAGCTAGAAATAGCAAG
sgRNA11-4fw	GAAATTAATACGACTCACTATAGGTCCCGGAAAAGGTCACCGTGTTTTAGAGCTAGAAATAGCAAG
sgRNA-23fw	GAAATTAATACGACTCACTATAGGACTTTGTATCGGTCTCGCGGTTTTAGAGCTAGAAATAGCAAG
sgRNA-124rv	GAAATTAATACGACTCACTATAGGCCTGCTGGTTCCAATAGCTGTTTTAGAGCTAGAAATAGCAAG
T7common	AAAAGCACCGACTCGGTGCCACTTTTTCAAGTTGATAACGGACTAGCCTTATTTTAACTTGCTATTTCTAGCTCTAAAAC
-0.6 CAD F	GGTGGAATTCTCCTTGGAAACATTT
+0.3 CAD R	CCCTCACTGTACATCGATGGC
-0.3 mALP F	GACTACTTCGGAACATAGGGCAAC
+0.3 mALP R	CTCTGGGCCTCGACAGTTAAG
CAD-F Exon3	ATAGCGGTCACTATTCCTGC
CAD-R Exon3	CATTGAAACTTCCTGTTCGCG
Nix F1	GATTTTTGTTTTTGTCGTGCAA
Nix R1	AATGCAAGTATCATAGGTCAGCA
rps3Ae Fwd	GGCATGTTCCGTGCTGAATTGAACG
rps3Ae Rev	TTCTCGGCGTACAGCTCGACG

### Embryo microinjection

Freshly laid eggs of *A*. *aegypti* were lined up, fixed with double-sided tape on a microscope slide and covered with halocarbon oil 700 (Sigma Aldrich). Microcapillaries of aluminosilicate glass were generated in the P-97 Micropipette puller (Sutter Instrument) with the setting parameters: heat = 548, pull = 60, velocity = 60, delay = 90, pressure = 300; then they were beveled with the MicroGrinder EG401 (Narishige) and filled up with a mixture containing 300 ng/μl of Cas9 nuclease NLS (Dharmacon Reagents) and two sgRNAs (125 ng/μl each). Approximately 0.5 nl of this mixture Cas9/sgRNAs were injected in each egg using the Micromanipulator MM3301-R (World Precision Instrument) and the Pneumatic PicoPump PV830 (World Precision Instrument). The halocarbon oil was removed carefully, and eggs were incubated in a humidity chamber for 4 days. Afterward, the eggs were submerged in ddH_2_O for embryos hatching.

### PCR and RT-PCR

Genomic DNA (gDNA) from the whole insect (adult or larvae) was isolated with the Quick-DNA Miniprep Plus kit (Zymo Research). Subsequently, the gDNA was used for PCR amplification of a DNA fragment containing the hybridization sites for sgRNAs by using Phusion Hight-Fidelity DNA polymerase and specific primer pairs. The -0.6 CAD F/+0.3 CAD R primes and CAD-F Exon3/CAD-R Exon3 primes were used for PCR amplification of internal *aae-cad* gene fragments of 721-bp and 160-bp, respectively. For amplification of the 985-bp *m-alp* gene internal fragment, we used -0.3 mALP F/+0.3 mALP R primers (Table 1). For RT-PCR assays, the total RNA was isolated from individual larvae of *A*. *aegypti* with RNeasy Kit (QIAGEN). The cDNA was synthesized from 0.5 μg of total RNA using a SuperScript III reverse transcriptase kit (Invitrogen Life Technologies). The ribosomal protein S3 (*rps3*) gene was used as housekeeping control and the *nix* gene was used as a genetic marker to identify the sex of the insects during larval instars (Table 1).

### *In vitro* Cas9 cleavage

CRISPR-Cas9-induced INDELs were identified through an *in vitro* assay that analyze the cleavage of DNA fragments induced by Cas9 nuclease. Briefly, a reaction mixture containing Cas9 nuclease (30 ng/μl), sgRNA (125 ng/μl each), corresponding DNA-target fragment (200 ng) obtained by PCR and Buffer3.1 1X (NewEngland BioLabs) was incubated for 1 h at 37°C. Then, the reaction was stopped by 10 min incubation at 75°C and analyzed by DNA electrophoresis in 1% agarose gel. Samples containing DNA that remained uncleaved or exhibited partial cleavage were chosen for DNA sequencing to validate the presence of INDELs.

### Selection of homozygous mutants

Microinjected embryos (G_0_) were reared to pupa. Each pupa G_0_ was isolated in a 20 ml plastic cup for hatching and adults were outcrossed with 2–3 wild-type adult mosquitoes of the opposite sex. Once the female laid eggs (G_1_), the selected adult mosquito G_0_ was genotyped as described above. If the G_0_ individuals presented INDELs, their resulting G_1_ offspring was reared. Subsequently, 4–6 larvae from this generation were pooled for genotyping, aiming to detect mutations that will indicate the presence of germline mutations. The G_1_ mutants were subjected to incross mating in pairs, and the resulting G_2_ offspring from couples harboring equivalent mutations were reared and subjected to incross mating in mass. The procedure was repeated for subsequent generations (G_2_ to G_4_ or more) until the establishment of the homozygous mutant.

### Enzymatic activity assays and ELISA

Specific enzymatic activity of ALP or APN were determined as was reported previously [21]. Briefly, the gut tissue from fourth instar larvae were dissected and homogenized. Then, protein concentration of gut tissue was estimated by the DC protein dye method (Bio-Rad) using BSA as standard. The ALP and APN activities were determined using *p*-nitrophenyl phosphate and l-leucine-*p*-nitroanilide as substrates, respectively. ELISA assay was performed to detect the Aae-Cad protein using the rabbit polyclonal antibody raised with a recombinant fragment G10 from the extracellular region of Aae-Cad [9]. For this assay, guts were dissected and the rest of the larvae body were used for gDNA isolation to sex them by PCR as was described above. Membrane proteins from twenty gut tissues from female or male larvae were extracted independently with *n*-Octil-β-D-Glucopyranoside 1% and immobilized on ELISA plates. Detection was performed with an anti-rabbit IgG-HRP antibody and ortho-phenylenediamine as substrate. The reaction was stopped adding H_2_SO_4_ and absorbance was measured at 495 nm.

### Production of Cry toxins and bioassay

Non-crystalliferous *B*. *thuringiensis* strain 4Q7 was transformed with the plasmids pCG6-Cry11Aa, pYG1-Cry11Ba, pHT606-Cry4Aa or pHT618-Cry4Ba to produce the crystal inclusion of Cry11Aa, Cry11Ba, Cry4Aa and Cry4Ba, respectively. The four strains were growth in HCT-agar medium supplemented with 10 μg erythromycin/ml at 30°C for 72 h until complete sporulation. The biomass was harvested and washed three times by centrifugation at 4°C to 12,000 *g* for 10 min in wash buffer (0.3 M NaCl, 1 mM EDTA, pH 8.0) and three times with dH_2_O. Protein concentration of the spore/crystal mixture was estimated by the Bradford method using BSA as protein standard. The toxicity assay was performed by suspending the mixture spore/crystal in 100 ml of dH_2_O containing 10 fourth-instar *A*. *aegypti* larvae. To estimate the concentration of Cry toxin that kill 50% of larvae (LC_50_) different doses of each Cry toxin were used and mortality was registered after 24 h. The LC_50_ value was calculated using a Probit analysis with the PoloPlus software (LeOra Software Company).

## Results

### KO mutation of the membrane-bound ALP in *A*. *aegypti* (Aae-mALP)

**Fig 1:**
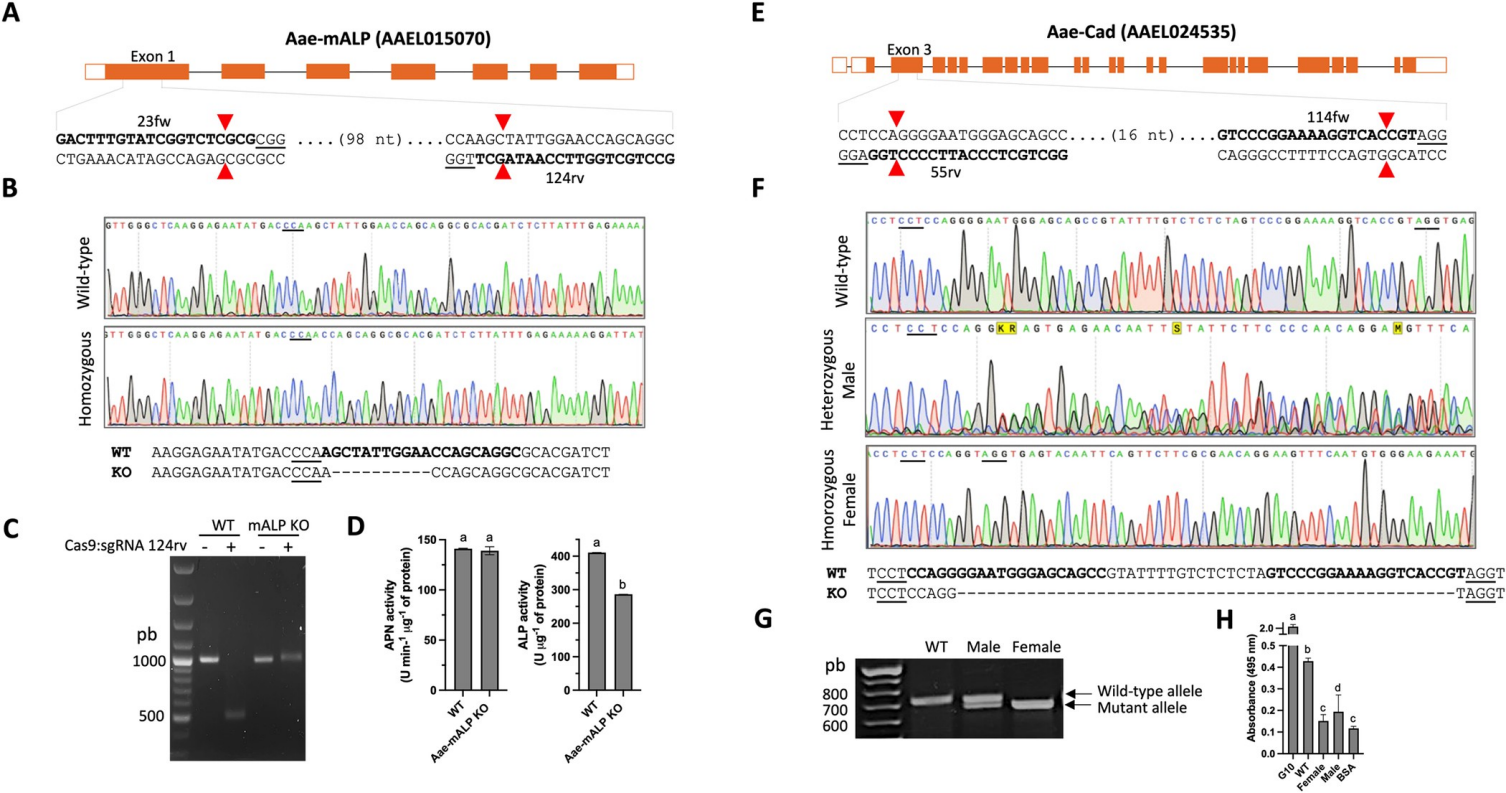
CRISPR-Cas9-mediated mutagenesis of *A*. *aegypti* Aae-mALP and Aae-Cad. **A**) Schematic representation of *aae*-*malp* gene targeted with two sgRNAs (23fw and 124rv) at the first exon. **B**) DNA sequencing chromatograms of Aae-mALP. The top sequence is Aae-mALP wild-type and bottom sequence correspond to homozygous mutant with 10-nt deletion. The alignment of the two sequence is showed at bottom. **C**) *In vitro* Cas9 cleavage assay of PCR products of *aae*-*malp* wild-type [44] and homozygous mutant Aae-mALP KO. Cas9:sgRNA-124rv digestion of the 985-bp PCR product results in two fragments of 491-bp and 494-bp. The cleavage of PCR product from Aae-mALP KO is impaired due to 10-bp deletion. **D**) Specific APN and ALP activities were determined from total proteins of fourth instar larvae gut tissues. **E**) Schematic representation of *aae-cad* gene. The third exon of this gene was targeted with 55rv and 114fw sgRNAs. **F**) DNA sequencing chromatograms of *aae-cad* wild-type (top chromatogram), heterozygous males (middle chromatogram) and homozygous mutant females (bottom chromatogram). The lower alignment shows the DNA sequences of wild-type and mutant female with 50-nt deletion. **G**) Detection of *aae-cad* alleles from Aae-Cad^(F)^ KO by PCR. PAM sequences are underlined, Cas9 cleavage-sites are indicated in red arrows and hybridization sites for sgRNAs are indicated in bold letters. **H**) ELISA assay for Aae-Cad detection from total proteins of fourth instar larvae gut tissues. The recombinant protein G10 that correspond to the extracellular region of Aae-Cad was used as positive control. Error bars are medians with SD. One-way ANOVA analysis among the analyzed groups is showed with letter in the top of bars.

We employed the CRISPR-Cas9 system to induce mutations in Aae-mALP, which was previously identified as a putative receptor for Cry4Ba toxin in *A*. *aegypti* [18]. The encoding gene for Aae-mALP protein (AAEL015070) contains seven exons (Fig 1A). We selected the first exon to design the sgRNAs using the CRISPOR server. Among multiple candidates, two sgRNAs located on opposite strands for targeting *aae*-*malp* gene were selected: 23fw and 123rv. Three-hundred eggs were injected with a mixture containing Cas9 nuclease and the two designed sgRNAs. A total of 16 hatched larvae (G_0_) were recovered after injection, all of them were reared to adulthood and outcrossed with wild-type mosquitoes from the opposite sex. A PCR fragment of 985-bp, spanning the sgRNAs hybridization sites of *aae-malp*, was obtained from the selected G_0_ adult insects. None of the candidates presented an evident PCR fragment with lower size in comparison to the wild-type gene, thereby we performed a screening of INDELs by the Cas9 cleavage assay as was described above and confirmed by DNA-sequencing. DNA-sequencing of PCR fragments showed overlapping peaks at the sgRNA hybridization region, indicating mutations mediated by CRISPR-Cas9. Off-spring of those insects (G_1_) with INDELs were further analyzed and distinct types of mutations were identified. For establishing a homozygous KO mutant an insect with a deletion of 10-bp located at the hybridization site of sgRNA-123rv (Fig 1B) was selected. A protocol of incross-mating by pairs was followed, making twelve couples per generation that were genotyped once females laid eggs. After the fourth generation, we obtained the homozygous population, hereafter named as Aae-mALP KO population. Finally, the mutation was confirmed by the *in vitro* Cas9 cleavage assay, where the mutated PCR fragment showed impaired cleavage due to the 10-bp deletion weakened the hybridization of sgRNA-123rv (Fig 1C). To further examine the phenotype of Aae-mALP KO, the specific activity of ALP was determined from epithelial tissue of Aae-mALP KO larvae. Data show that enzymatic activity of ALP from Aae-mALP KO larvae was reduced by about 30% compared with wild-type larvae. In contrast, the APN activity, which is another enzyme associated to epithelial tissue, was similar than wild-type larvae (Fig 1D).

### Mutations in cadherin (Aae-Cad) are sex-linked in *A*. *aegypti*

In the case of the Aae-Cad KO, we followed a similar approach as described above. The encoding gene *aae-*c*ad* (AAEL024535) contains 23 exons, and two sgRNA (55rv and 114fw) were designed for targeting the third exon of this gene (Fig 1E). After injection of 300 eggs, 22 embryos hatched and a mutant allele with a 50-bp deletion flanked by the hybridization sites of the two sgRNAs was selected (Fig 1F). Over four generations, we obtained homozygous mutants exclusively in female insects, while males consistently exhibited a heterozygous genotype (Fig 1F and 1G). This distinctive pattern persisted over the subsequent six generations (up to G_10_). The mutant population was designated as Aae-Cad^(F)^ KO, indicating its association with females (a 50-bp deletion in *aae-cad* gene). An ELISA assay was performed to identify the presence or absence of Aae-Cad protein in males or females from the Aae-CAD^(F)^ KO population using a polyclonal anti-Aae-Cad antibody [9]. For this assay, fourth instar larvae were initially sexed using PCR to detect the *nix* gene located at the *M-locus* of male individuals [22] and then gut tissue membrane proteins were used for Aae-Cad detection. Surprisingly, results showed that males presented a lower level of Aae-Cad protein than the wild-type, possibly attributable to the presence of a single edited *aae-cad* allele, while Aae-Cad was not detectable in females since ELISA signal was indistinguishable from the BSA control (Fig 1H).

**Fig 2:**
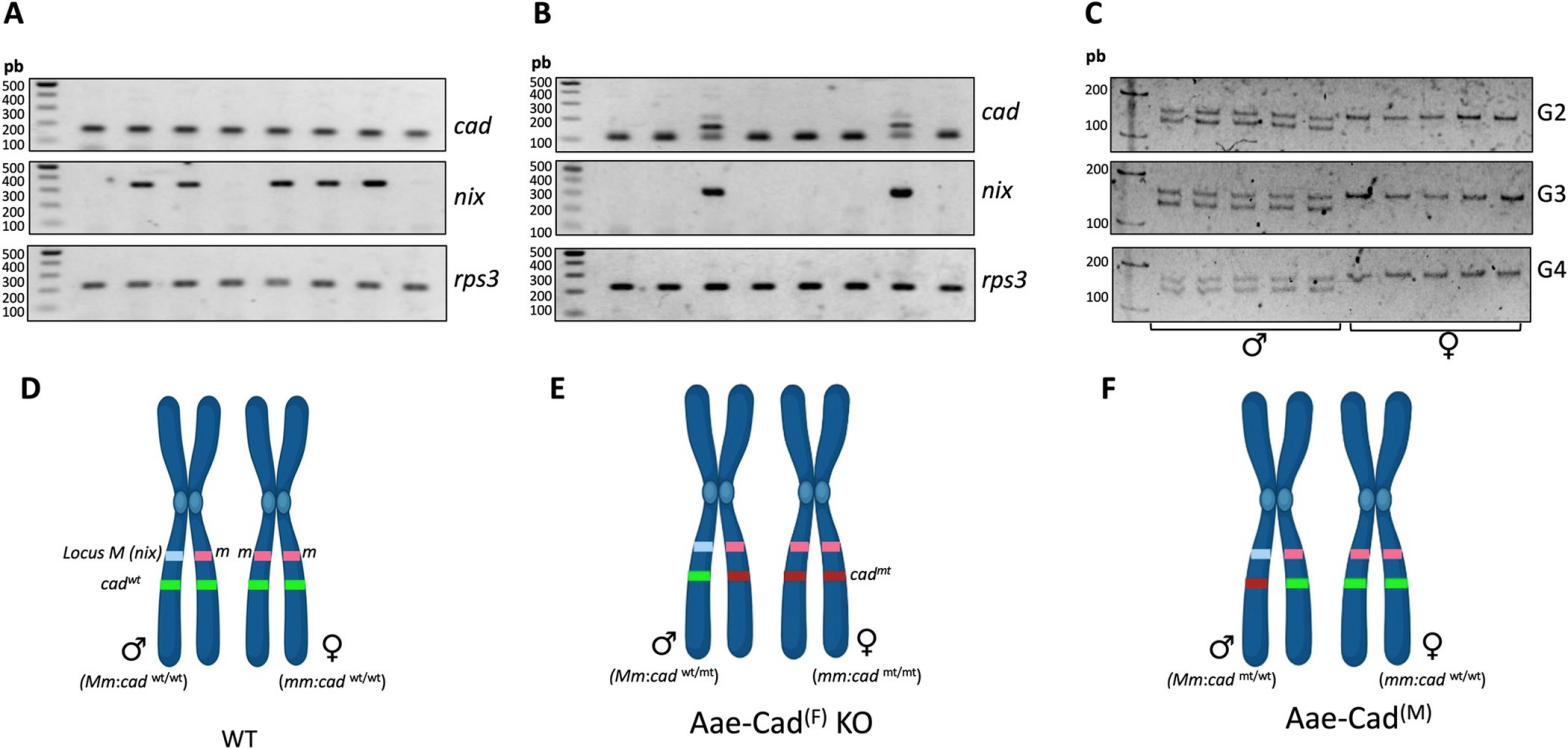
Sex-linked behavior of Aae-Cad mutations. RT-PCR analysis of eight fourth-instar larvae randomly selected from wild-type (**A**) and Aae-Cad^(F)^ KO (**B**) populations. Among eight Aae-Cad^(F)^ KO larvae, two were males (*nix* positive) and presented both *aae-cad* alleles, the wild-type and mutant with 50-nt deletion, while the six females (*nix* negative) presented only the allele with 50-nt deletion. The *rps3* gene was used as a housekeeping reference. **C**) PCR analysis of ten adults from Aae-Cad^(M)^ KO population from different generations (G2 to G4). PCR products of five males and five females from each generation were analyzed in 6% polyacrylamide gel and the 10-bp deletion was observed only in one *aae-cad* allele of males. **D**) Scheme of chromosome 1 with the loci (*M*:*m)* in males and (*m*:*m*) in females. The location of *aae-cad* gene is showed with a green line. **E**) In the case Aae-Cad^(F)^ KO population, the 50-bp mutation in *aae-cad* (red line) was generated in the chromatid associated to *m*-locus and only females are homozygous mutant. **F**) The 10-bp deletion in *aae-cad* (red line) generated in Aae-Cad^(M)^ KO population is associated to *M*-locus, thereby only males inherit the mutation being heterozygous.

A detailed analysis of *aae-cad* expression by RT-PCR during the larval fourth instar stage was conducted. Eight fourth-instar larvae were randomly selected and subjected to RT-PCR sexing, which involved the detection of the *nix* gene located at the *M-locus* of male individuals [22]. As expected, larvae lacking the *nix* gene (females) expressed only the mutant allele of *aae-cad* with the 50-bp deletion, while *nix*-positive larvae presented both mutant and wild-type alleles (Fig 2A and 2B). These findings indicate that males of *A*. *aegypti* Aae-Cad^(F)^ KO population contain a non-recessive *aae-cad* wild-type allele. Interestingly, the selected G_1_ insect with the deletion of 50-bp were females. Therefore, we decided to analyze a different type of mutation found in a G_1_ male insect with a deletion of 10-bp located at the hybridization site of sgRNA-55rv. In contrast to the lineage Aae-Cad^(F)^ KO, we observed that over four generations only males consistently were heterozygous containing the mutant and wild-type alleles (named as Aae-Cad^(M)^ population), while the females maintained the wild-type alleles (Fig 2C). Over the next six generations (up to G_10_), ten females per generation were analyzed, and none of them presented the 10-bp deletion (data not shown). Thus, we were unable to establish the homozygous Aae-Cad^(M)^ mutant population across 10 generations.

To gain insights into the behavior of sex-linked mutations in *aae-cad* gene, we identified its genomic location. *A*. *aegypti* genome is composed by three chromosomes, and the *aae-cad* gene is located in chromosome 1. Notably, this chromosome harbors the (*M*:*m*) *loci*, recognized as the Y chromosome-like region. The *M-locus* determines the sex in males, bearing a heterozygous genotype (*M*:*m*), while females have a homozygous genotype (*m*:*m*) [22]. Interestingly, *aae-cad* and the (*M*:*m*) *loci* are located in the same chromatid (Fig 2D). This explains that the deletion of 10-bp in *aae-cad* gene occurred in the chromatid containing the *M-locus*, resulting in the lineage Aae-CAD^(M)^ with heterozygous males (Fig 2E). In contrast, the 50-bp deletion mutation occurred in the non-sister chromatid containing the *m-locus*, generating the lineage Aae-CAD^(F)^ KO with a heterogeneous population comprising heterozygous males and homozygous mutant females (Fig 2F).

### Development of *A*. *aegypti* is not affected by the Aae-mALP or Aae-Cad^(F)^ KO mutations

**Fig 3:**
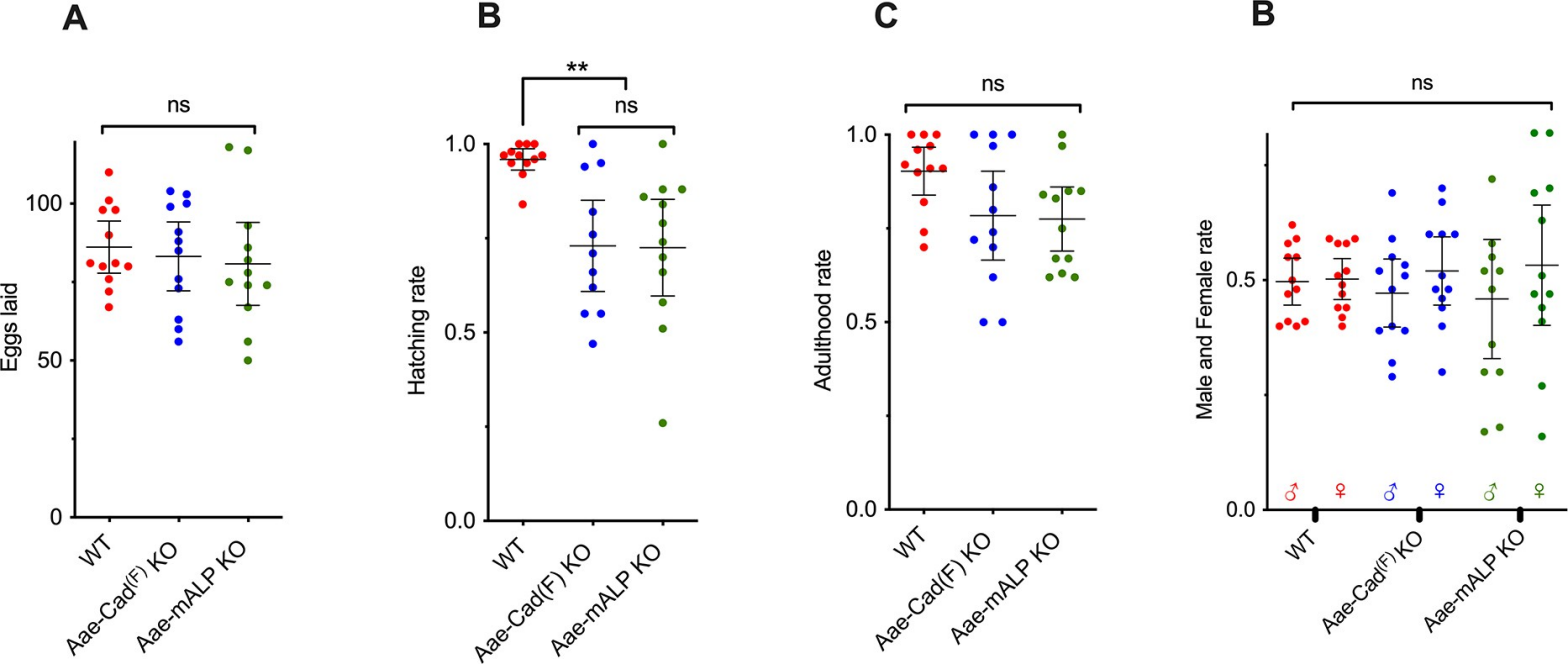
Development analysis of *A*. *aegypti* mutant populations. Twelve females were isolated after blood feeding and monitored for eggs laid (**A**), hatching (**B**), development to adulthood (**C**) and differentiation to males (♂) and females (♀) (**D**). Error bars are medians with 95% confidence intervals. One-way ANOVA analysis was performed to obtain the *P* values. Values of *P* > 0.05 were considered not statistically significant (ns) and *P* ≤ 0.01 are statistically significant (**).

We further analyzed whether Aae-mALP KO and AaeCad^(F)^ KO mutant populations were affected in their development. For this purpose, the *A*. *aegypti* populations were blood fed and females were separated to analyze their fertility. The comparison between the two KO mutant and wild-type populations showed that there were no significant differences in egg laid (P > 0.05) (Fig 3A). However, there was a significant difference in egg hatching, since Aae-mALP KO and Aae-Cad^(F)^ KO mutants exhibited 23% lower hatching rate than *A*. *aegypti* wild-type population (Fig 3B). Aae-mALP KO and Aae-Cad^(F)^ KO larvae were reared to adulthood showing a similar survival rate as the wild-type population (Fig 3C). Finally, in order to analyze if the mutant populations present a tendency for a specific sex differentiation, the rate of males and females from each population was analyzed. The data showed a slight tendency of females for Aae-mALP KO and Aae-Cad^(F)^ KO populations, but statistical test revealed that these data were no significant different (Fig 3D). All these parameters indicate that the KO mutations of Aae-mALP and Aae-Cad did not have an evident effect on the development of these two mutant populations of *A*. *aegypti*.

### Susceptibility of Aae-mALP and Aae-CAD^(F)^ KO populations to the mosquitocidal Cry toxins

The susceptibility of Aae-mALP KO and Aae-Cad^(F)^ KO populations to the insecticidal Cry toxins, Cry4Aa, Cry4Ba, Cry11Aa and Cry11Ba, was tested by bioassays. Different concentrations of Cry toxins were used to estimate the LC_50_ values. The LC_50_ values of Aae-mALP KO population for Cry11Aa, Cry11Ba, Cry4Ba and Cry4Aa were 490.50 ng/ml, 532.15 ng/ml, 873.78 ng/ml and 1106.07 ng/ml, respectively (Table 2). The LC_50_ values obtained with the *A*. *aegypti* wild-type population were similar since 95% fiducial limits overlapped, suggesting that the KO of Aae-mALP did not confer resistance to the Cry toxins tested. Similar results were observed when the susceptibility of Aae-Cad^(F)^ KO population was tested against the same Cry toxins. The LC_50_ values for both the *A*. *aegypti* wild-type and Aae-Cad(F) KO populations are shown in Table 2.

**Table 2:** Susceptibility of Aae-mALP KO and Aae-Cad^(F)^ KO *A*. *aegypti* populations to Cry toxin.

	Toxin			
	Cry11Aa	Cry11Ba	Cry4Ba	Cry4Aa
**WT**	367.16 (226.4–482.3)	484.15 (351.7–578.6)	829.41 (715.4–975.6)	1032.63 (569.3–1263.1)
**Aae-Cad**^**(F)**^ **KO**	490.50 (326.2–645.6)	532.15 (287.7–653.5)	873.78 (639.0–1058.1)	1106.07 (845.3–1303.6)
**Aae-mALP KO**	549.43 (432.3–663.5)	621.94 (480.7–805.5)	861.52 (735.0–1017.5)	1025.60 (860.3–1185.0)

The values are the concentration of the toxin killing the 50% of insects (LC_50_) expressed in ng/ml, and parenthesis show the 95% fiducial limits. Bioassays were carried out in triplicate.

**Fig 4:**
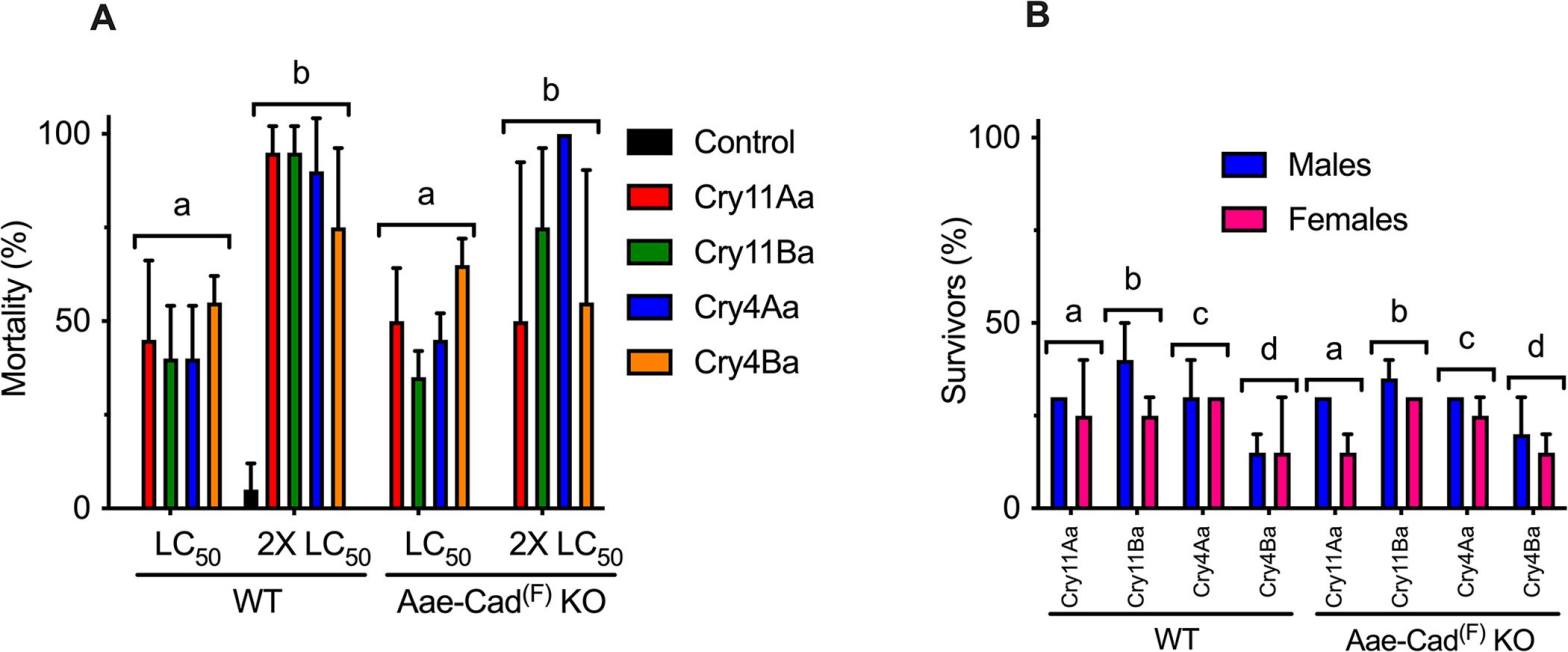
Bioassays. Fourth-instar larvae from *A*. *aegypti* wild-type and Aae-Cad(F) KO populations were exposed to LC_50_ or 2-fold the LC_50_ of Cry toxins and mortality was registered after 24 h (A). Larvae that survived were reared to adulthood and the percentage of males and females was registered (B). Error bars are medians with SD. One-way ANOVA analysis among the analyzed groups is showed with letter in the top of bars.

It is noteworthy that the Aae-Cad^(F)^ KO population is heterogeneous, since only females possess a complete KO of Aae-Cad, while males carry one unedited *aae-cad* allele. Therefore, we carried out bioassays using the LC_50_ dose and 2-fold the LC_50_ (Fig 4A). Larvae were exposed to the different Cry toxins during 24 h and then survivors of to these toxin treatments were reared to adulthood. We hypothesized that if the KO of Aae-Cad confers resistance, the KO-females would survive to Cry toxin treatment. Since the resistance ratio is defined as the LC_50_ of resistant insects divided by the LC_50_ of susceptible insects, the minimum RR expected for a resistant strain is a value of 2. The results showed that either males or females survived to the toxin treatment with no significant differences (Fig 4B), supporting that the KO of Aae-Cad in the females does not confer resistance to Cry toxins.

## Discussion

*B*. *thuringiensis* Cry toxins are a group of insecticidal proteins that specifically target insect species during their larval instars. Several insect-membrane proteins have been identified as potential receptors for Cry toxins influencing their specificity. In the mosquito *A*. *aegypti*, Aae-Cad and Aae-mALP are membrane proteins that exhibit high-affinity binding to Cry11Aa or Cry4Ba toxins, respectively [9,23]. The remarkable high affinity in these interactions suggests their potential role as receptors for these Cry toxins. In several insect pests, Cad-like proteins have been described as receptors for Cry toxins. Studies performed with lepidopteran insects have clearly shown that Cad is a functional receptor for certain Cry1 toxins such as Cry1Ac in specific insect species as *H*. *virescens*, *P*. *gossypiella* and *H*. *armigera* [5–7], while it is irrelevant for other toxins such as Cry1Fa in other insect species as *S*. *frugiperda* and *S*. *exigua* [24,25]. Cad are localized at the apical side of gut epithelium cells. Although their precise physiological role in the midgut cells is still unknown, it has been proposed that they contribute to the structural integrity of the microvilli. Several reports with lepidopteran insects, have showed that the absence of Cad does not have a remarkable fitness cost and showed a correlation with resistance to some Cry toxins [5–7]. In the case of dipteran insects, a recent study aimed to generate an Aae-Cad KO population in *A*. *aegypti* using a range of genetic editing tools such as TALENT, ZFN and CRISPR-Cas9, showed that it was not possible to generate an Aae-Cad KO strain. The authors concluded that Aae-Cad might be essential for *A*. *aegypti* development [26]. Here, we were persistent to generate the Aae-Cad KO in *A*. *aegypti*, since previous works have successfully generated the Cad KO mutations in lepidopteran species using CRISPR-Cas9 technology and the role of this protein as a functional receptor for Cry toxins has been demonstrated [24,25,27,28]. Our CRISPR-Cas9 KO mutations in the *aae-cad* gene resulted in the generation of premature stop codons in the mRNA. Interestingly, the genetically modified *A*. *aegypti* mosquitoes with KO mutations in *aae-cad* showed a sex-linked genotype. This phenomenon could be attributed to the proximal positioning of the *aae-cad* gene with the *loci* (*M*:*m*) on chromosome 1, which is the sex-determining factor in *A*. *aegypti* [22]. A particular characteristic of the *M-locus* is its documented lack of chromosomal recombination events [29], which would explain why the 10-bp and 50-bp deletions in *aae-cad* genes from the Aae-Cad^(M)^ and Aae-Cad^(F)^ populations, respectively, did not presented a chromosomal recombination after more than 10 generations. Consequently, this absence of recombination with the “non-sister chromatid” has prevented the generation of isogenic mutant populations. However, we have successfully generated females *A*. *aegypti* with homozygous Aae-Cad^(F)^ KO mutation that exhibited normal development. Our data could explain the finding of Chen et al., (2020), who exclusively observed heterozygous insects over eight generations and they were unable to stablish a homozygous Aae-Cad KO population. This observation could be attributed to the omission of a thorough analysis regarding the correlation between Aae-Cad mutations and insect sex in their study [26]. The sex-linked behavior of DNA mutations located in the chromatid containing the *M-locus* have been observed previously. One of the classic examples is the *myo-sex* gene, which is located within the *M-locus* alongside with the *nix* gene. The *myo-sex* gene encodes for the myosin heavy-chain, and its KO is associated with a flightless phenotype exclusively in males [30]. Initially, it was proposed that the *M-locus*, containing the *nix* and *myo-sex* genes, is a non-recombinant region of 1.28 Mbp, known as sex-determining region (SDR). However, recent studies have shown that the SDR is much larger, estimating a size of 123 Mbp [31], which would include the *aae-cad* gene. This hypothesis was tested using two genetic markers to estimate the recombination rate of the chromatid containing the *M-locus*. The proximal region to the *M-locus* (where the *aae-cad* gene is located) showed a recombination rate of 1.0 to 1.5 cM, while the distal region had a rate of 2 to 3 cM, indicating null or very low recombination rate along the whole sex-determining chromatid [32]. All these studies clearly show that the chromatid containing the *M-locus* is exclusively inherited to males and it has a very low recombination rate, which support the sex-linked behavior of mutations in *aae-cad*.

The Aae-Cad protein is localized on the apical membrane of the cells found in the caecae and posterior midgut regions of fourth-instar *A*. *aegypti* larvae. These regions correspond to Cry11Aa binding regions in the mosquito midgut tissue, to which Cry11Aa binds with a high affinity of ~16 nM [9,26]. Significant studies have demonstrated that Aae-Cad serves as a functional receptor for some Cry toxins *in A*. *aegypti*. When the Aae-Cad protein was expressed in the *A*. *albopictus* C6/36 cell line, it exhibited moderated sensitivity to Cry11Aa, Cry11Ba, and Cry4Aa but not to Cry4Ba [33]. This observation is consistent with the lower binding affinity of ~135 nM between Cry4Ba and Aae-Cad [12]. Further investigations, involving larvae with silenced Aae-Cad expression by dsRNAi, revealed tolerance to Cry11Aa but not to Cry4Ba [12,33]. All these data suggest that Aae-Cad could be receptor for Cry11Aa, Cry11Ba, and Cry4Aa. However, our results in this study showed that the KO populations of Aae-Cad^(F)^ KO generated by CRISPR-Cas9, did not change their susceptibility to mosquitocidal Cry toxins, suggesting that Cad is not playing a major role in the mechanism of action of Cry toxins in *A*. *aegypti*. These results correlated with the transcriptomic study of four *A*. *aegypti* resistant populations to Cry11Aa, Cry4Ba, and Cry4Aa or Bti crystals, where no specific up/down regulation was observed in none of the twelve Cad-like proteins [34–36]. The authors concluded that Cad might not have a central role in resistance to Bti in mosquitoes suggesting that other proteins may be implicated in the resistance to these Cry toxins in *A*. *aegypti* [36].

Regarding Cry4Ba receptors, similar approaches were conducted to identify and confirm their functional roles. One of these receptors is Aae-mALP, which has been identified as a binding protein for Cry4Ba with a binding affinity of ~80 nM [16,23]. When the *S*. *frugiperda* Sf9 cell line expressed the Aae-mALP, this transformed cell-line exhibited moderated susceptibility to Cry4Ba [17], suggesting that Aae-mALP serves as receptor for Cry4Ba. However, our results in this study showed that the Aae-mALP KO mutation generated by CRISPR-Cas9, does not change their susceptibility to Cry11Aa, Cry11Ba, Cry4Ba and Cry4Aa toxins. It is noteworthy to mention that the *A*. *aegypti* genome harbors 11 ALPs that may potentially substitute the receptor function of Aae-mALP in the Aae-mALP (KO) population. Among these ALPs, the Aae-ALP1 (AAEL009077) was shown to bind Cry11Aa [13], while Aae-ALP2 (AAEL000931) and Aae-ALP3 (AAEL003286) bind to Cry11Ba [37]. Therefore, further studies targeting these additional ALPs should be conducted to elucidate their role in the mode of action of Cry toxins.

It is intriguing that silencing the expression of Aae-Cad resulted in tolerance to Cry11Aa [12], but Aae-Cad KO mutations showed no resistant phenotype. A possible explanation is that lowering the expression of Aae-Cad by dsRNAi does not result in the expression of additional proteins that compensate the lack of Cad production. In contrast, in the Aae-Cad KO mutant the compensatory expression of other midgut proteins is necessary to compensate the lack of Cad expression which could also participate as Cry-receptors masking the phenotype of Cry toxin resistance. In a previous work, the expression of different putative Cry-receptors was analyzed in three *A*. *aegypti* resistant strains, that were selected against individual toxins Cry4Aa, Cry4Ba and Cry11Aa that showed 1018-, 226- and 70-folds resistance, respectively. The analysis of expression of different putative Cry receptors, ALPs, APNs, and α-amylases, glycoside hydrolase and ABC transporter, revealed that ALPs and α-amylases were overexpressed in the *A*. *aegypti* resistant strains to Cry4Aa and Cry11Aa, respectively [35]. Similarly, in the Cry1Ac-resistant *Trichoplussia ni*, APN1 was significantly down-regulated, whereas APN6 was significantly up-regulated, hence overexpression of certain protein isoforms compensate the reduction of other protein isoforms [38]. It is possible that KO mutations of specific proteins may result in compensatory effects in the expression of other proteins. An additional explanation could be that some proteins may have redundant roles as Cry-receptors. It has been suggested that APN and ALP receptors may exhibit a redundant role in *M*. *sexta* [39], thus both proteins should be mutated in order to observe a resistance phenotype. Similarly, it has been shown that ABCC2 and ABCC3 receptors of the lepidopteran *H*. *armigera* and *P*. *xyllostella* have redundant functions as receptors for Cry1A toxins since ABCC2-KO mutations showed low resistance levels and only double ABCC2-ABCC3-KO strains showed very high Cry1A-resistance levels, indicating that ABCC2 and ABCC3 have redundant roles in toxicity [40,41]. Also, in *A*. *aegypti* two APNs have also been identified as receptors for Cry11Aa toxin [42]. However, individual or simultaneous CRISPR-Cas9 KO of these two APN in *A*. *aegypti* does not affected the susceptibility to Cry11Aa and Cry4Ba toxins suggesting that additional APN isoforms could compensate the lack of expression of the edited *apn* genes [43].

In summary, the results presented here showed that individual KO mutations of Aae-Cad and Aae-mALP does not change the susceptibility of *A*. *aegypti* to the Cry toxins produced by Bti. The Aae-Cad KO was sex-linked but does not affect the development of *A*. *aegypti* mosquitoes. Given that our approach involved the use of CRISPR-Cas9 to generate a genetically modified organism serving as an *in vivo* model, our data indicate potential influence of additional molecules on the activity of Cry toxins or that a redundant role among receptors may be implicated in Cry toxin action in mosquitoes.
